# West Nile outbreak in horses in southern France, 2000: the return after 35 years.

**DOI:** 10.3201/eid0704.010417

**Published:** 2001

**Authors:** B Murgue, S Murri, S Zientara, B Durand, J P Durand, H Zeller

**Affiliations:** Centre National de Référence des Arbovirus et des Fièvres Hémorragiques Virales, Institut Pasteur-25, rue du Dr-Roux, 75724 Paris cedex 15, France. bmurgue@pasteur.fr

## Abstract

On September 6, 2000, two cases of equine encephalitis caused by West Nile (WN) virus were reported in southern France (Hérault Province), near Camargue National Park, where a WN outbreak occurred in 1962. Through November 30, 76 cases were laboratory confirmed among 131 equines with neurologic disorders. The last confirmed case was on November 3, 2000. All but three cases were located in a region nicknamed "la petite Camargue," which has several large marshes, numerous colonies of migratory and resident birds, and large mosquito populations. No human case has been confirmed among clinically suspected patients, nor have abnormal deaths of birds been reported. A serosurvey has been undertaken in horses in the infected area, and other studies are in progress.


### West Nile Outbreak in Horses in Southern France, 2000: The Return after 35 Years

**Bernadette Murgue,* Séverine Murri,* Stephan Zientara,† Benoît Durand,† Jean-Paul Durand,‡ and Hervé Zeller***

*Centre National de Référence des Arbovirus et des Fièvres Hémorragiques Virales, Institut Pasteur, Paris, France; †Agence Française d Sécurité Sanitaire des Aliments, Maisons-Alfort Cedex, France; ‡Institut de Médecine Tropicale du Service de Santé des Armées, Marseille Armées, France

On September 6, 2000, two cases of equine encephalitis caused by West Nile (WN) virus were reported in southern France (Hérault Province), near Camargue National Park, where a WN outbreak occurred in 1962. Through November 30, 76 cases were laboratory confirmed among 131 equines with neurologic disorders. The last confirmed case was on November 3, 2000. All but three cases were located in a region nicknamed "la petite Camargue," which has several large marshes, numerous colonies of migratory and resident birds, and large mosquito populations. No human case has been confirmed among clinically suspected patients, nor have abnormal deaths of birds been reported. A serosurvey has been undertaken in horses in the infected area, and other studies are in progress.

West Nile (WN) fever is a mosquito-borne flaviviral infection transmitted in natural cycles between birds and mosquitoes, particularly *Culex* species. In humans, WN infection is usually an asymptomatic or mild febrile illness; however, encephalitis cases are reported with some fatalities in older patients. WN virus is also a cause of animal disease, especially in horses.

WN virus was discovered in 1937 in the blood of a woman in the West Nile Province of Uganda who had a mild febrile illness (1). Since then, both sporadic cases and major outbreaks of WN fever in humans and equines have been reported in Africa, the Middle East, Europe, and Asia (2), and many aspects of WN infection have been well documented elsewhere since the early 1950s (3-7). During the last 5 years, many reports about WN virus have been published (8-17).

In France, the first reported outbreak occurred during the summer of 1962 in the Camargue region (Bouches-du-Rhône Province). At that time, several horses had neurologic disorders. As many of these horses were living wild, the exact number of animals with clinical symptoms was not known. However, among domestic horses for which information was available, 50 cases with neurologic signs, 25% to 30% of them fatal, were reported during the summer of 1962, with a peak between August 15 and September 15. The disease was mainly characterized by ataxia, weakness, and amaurosis (6). Several human cases of encephalitis were also reported during the same period in Camargue and Languedoc (Herault Province). However, no precise data were available for these patients except for one who was hospitalized with fever and meningitis and who had antibodies against group B arboviruses (18). WN virus infection could not be confirmed until 1964, when the virus was isolated in September from *Culex modestus* mosquitoes and the blood of two entomologists working in the field (19). Subsequently, 13 human patients, recorded from September 1962 to September 1964, were confirmed by hemagglutination-inhibition and neutralization tests to have infection compatible with WN virus (5), including one fatal case (September 1962). In 1963 and 1964, a serosurvey was conducted in 47 horses located in Camargue, including 10 animals who had neurologic signs in 1962. Neutralizing antibodies against WN virus were detected in 6 of 37 animals without clinical symptoms and 6 of 10 with previous disease (6). In 1965, WN virus infection was confirmed in three horses with neurologic signs, including one fatal case from which virus was isolated from the spinal cord. The same year, virus also was isolated from *Cx. modestus* mosquitoes (20).

After 1965, there was no evidence of WN virus infections in humans or horses. During a serosurvey (hemagglutination-inhibition assay) conducted in Camargue from 1975 to 1979, a low frequency of antibody response against WN virus was observed in 235 human samples (4.9%) and 99 horse samples (2%) (21). In contrast, a high frequency was observed against Tahyna virus (31% in humans and 9% in horses), a Bunyavirus belonging to the California group that induces febrile illness with central nervous system signs and has been reported in many countries in Europe as well as in Africa and Asia (22).

### Materials and Methods

### The Outbreak

On September 6, 2000, WN immunoglobulin M (IgM)-capture enzyme-linked immunosorbent assay (MAC-ELISA) and indirect IgG ELISA results were positive for two samples

Address for correspondence: Bernadette Murgue, Centre National de Référence des Arbovirus et des Fièvres Hémorragiques Virales, Institut Pasteur - 25, rue du Dr-Roux - 75724 Paris cedex 15, France; fax: 33-01-4061-3151; e-mail: bmurgue@pasteur.fr from horses. These horses were located in the same village in the south of France (Lansargues, Hérault), approximately 10 km from Montpellier (Figure 1). On August 24 and 28, they had signs of acute neurologic disorders, characterized by high fever and paresis of the hindquarters, then paralysis of the hind legs and inability to get up. The horses were euthanized on August 30 and September 1, respectively. Retrospectively, the same veterinary practitioner reported the case of a horse in the same village, which had clinical symptoms compatible with WN virus infection on August 3 and died 9 days later. WN infection was confirmed on September 8 by detection of WN viral RNA in a brain biopsy of one horse sampled for rabies diagnosis.

An alert was launched by both the ministry of health and the ministry of agriculture. Mosquito larvicide, targeted at *Cx. modestus* mosquitoes, was applied on September 9 to an area of about 200 ha near the confirmed cases. Restricted movement measures imposed by the Commission of the European Communities, were applied to equines within a 25-km radius area around a holding on which WN fever was confirmed in equines during the previous 30 days. These equidae were held for 21 days in isolation quarantine, after which MAC-ELISA was performed by a method derived from Zeller et al. (23). Briefly, IgM antibodies were captured with a goat anti-horse mu-chain antibody (Sigma Chemical Co., St. Louis, MO). WN antigen, prepared on Vero E6 cells and inactivated by beta-propiolactone, was added. Specific binding was demonstrated by using a WN mouse immune ascitic fluid and a peroxydase-labeled anti-mouse antibody. IgG antibodies in sera were detected by a method derived from Tsai et al. (11). Plates were coated with WN antigen, and IgG antibodies were revealed by a peroxydase-labeled anti-horse IgG antibody (Biosis, Compiègne, France). Sera were considered positive if the optical density was >3 standard deviations above the mean of negatives.

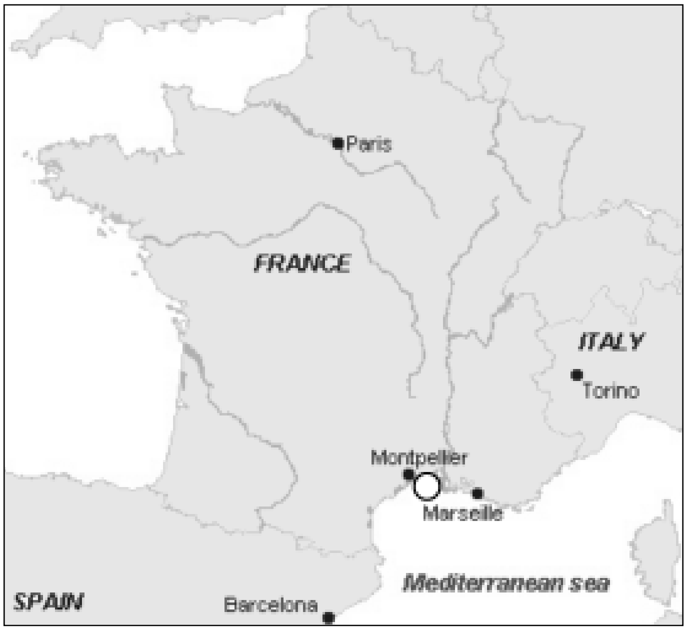

Figure 1. Geographic location of horses with laboratory-confirmed West Nile virus infection, France.*
*Open circle indicates location of confirmed cases.

### Results

As of November 30, we had received samples from 129 horses and 2 donkeys clinically suspected of having WN virus infection by veterinary practitioners (neurologic signs such as ataxia, paresis, or paralysis, with or without fever >38.5°C). A confirmed case was defined as illness in an equine with clinical suspicion of WN virus infection and a positive WN virus IgM antibody test result; a probable case had a negative WN virus IgM test result and a positive IgG antibody test result. A total of 58 equines were defined as having confirmed cases (57 horses and 1 donkey) and 18 horses as probable cases. Twenty (34%) of the animals with confirmed cases and one (6%) of the probable cases died. Eight of the 58 confirmed cases had a negative IgG antibody test result; 4 of these 8 died. Of the probable cases, two had samples obtained 15 and 23 days after illness onset; the rest had samples obtained during the acute phase of illness. The clinical symptoms of the confirmed and probable cases were similar, as was the age distribution of the animals (mean 12.5 ± 5.3 vs. 12.0 ± 6.6 years for confirmed and probable cases, respectively).

Most positive samples (confirmed and probable) were reported in September (82.9%). The last case was reported on November 3. The clinical symptoms included mainly fever (>38.5°C), ataxia, paresis, and paralysis (Table).

Ages of confirmed and probable cases ranged from 3 to 30 years (mean 12 years, median 10 years). There were 4 stallions, 20 mares, and 49 geldings (no information for 3 horses). Most fatal cases (57.1%) were recorded before September 15 (Figure 2); among fatal cases, 18 (86%) were euthanized, including one donkey that had neurologic signs

Table. Clinical features of disease in 76 horses with confirmed or probable West Nile virus infection

| Clinical signs | No. of horses (%) |
|---|---|
| Fever (>38.5°C) | 47 (62%) |
| Ataxia | 55 (72%) |
| Paresis/paralysis | 36 (47%) |
| Tremor | 7 (9%) |
| Hyperesthesia | 6 (8%) |
| Grinding teeth | 3 (4%) |
| Abnormal behavior | 2 (3%) |
| Hepatitis | 1 |

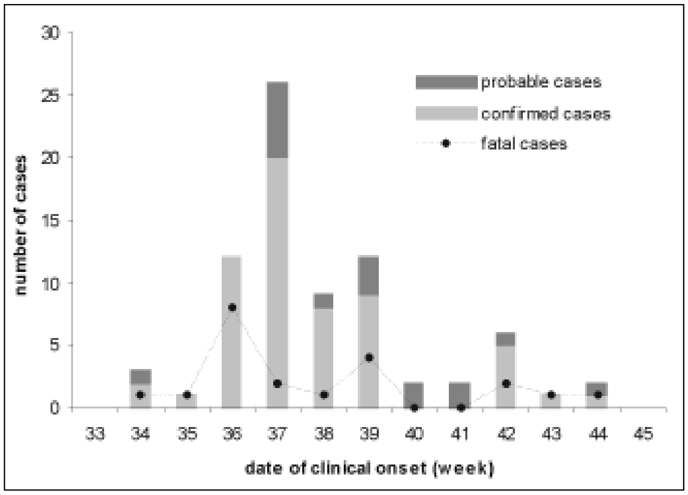

Figure 2. West Nile confirmed, probable, and fatal equine cases, by week of clinical onset, France.

followed by a short period of remission and then severe hepatic failure. Ages were not known for 4 of the 21 fatal cases. Of the 17 horses for which information was available, 41.2% and 29.6% were in the 6- to 10- and 16- to 20-year age categories, respectively (Figure 3).

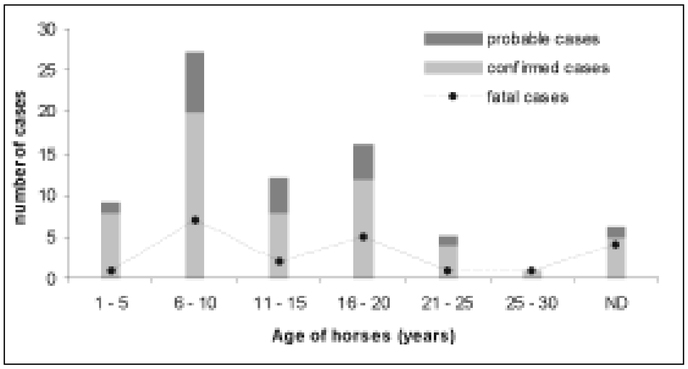

Figure 3. Age of horses with confirmed, probable, and fatal West Nile virus infection, France.*
*ND = not determined.

All but three confirmed and probable cases were located in an area within a radius of 15 km, in a region in Hérault and Gard provinces called “la petite Camargue.” Thirty-one (40.8%) of the horses were located within a 5-km radius of the first two reported cases (Lansargues). Three cases, all fatal, were located near this area, in Bouches du Rhone Province approximately 30 km from the first reported cases and 15 km outside the area where confirmed cases were reported. These animals, according to the owners, had not moved from this area during the 3 weeks preceding the onset of symptoms. However, because of the economic consequences of the restricted movement measures imposed by the Commission of European Communities, we assume some owners may not have observed the restrictions.

We also received 33 samples from other animals, some of them with neurologic signs, in the infected area during the outbreak: 16 cows, 8 goats, and 9 others (e.g., camel, dog, zebra). WN ELISA results were negative for all of them.

No human suspected of having WN infection has been laboratory-confirmed among 51 persons tested, including 33 hospitalized with signs of encephalitis or meningoencephalitis and 18 others with fever or living in close contact with horses. All these samples were obtained from persons living or traveling in the infected area during the outbreak. In contrast, WN IgG antibodies were detected in 3 of 33 gamekeepers working in this area. Two had WN neutralizing antibodies: one had no WN IgM antibodies; the other (who had no history of travel during recent years) had low but detectable IgM antibodies.

### Virus Isolation and Molecular Characterization of Virus Isolates

WN virus was isolated after one passage into C6/36 and Vero E6 cells from the rachidian bulb of the first confirmed case and from cerebellum, cortex, and lumbar spinal cord of another horse that died on September 6. Viral RNA was extracted from culture supernatants and a reverse transcription-polymerase chain reaction (RT-PCR) was performed with primers located in the envelope gene fragments WN240 and WN132, as described (24). Nucleic acid sequences were obtained on an automated Applied Biosystems sequencer (PPE Biosystems, Foster City, CA). WN virus sequences were aligned by using the multiple sequence alignment software CLUSTAL.

Phylogenetic analysis of an informative region of the E glycoprotein gene (Figure 4), using tree-view, showed that the WN France-2000 isolate belonged to lineage 1 and was closely related to both horse Morocco-1996 and Italy-1998 isolates. It is also closely related to mosquito isolates from Senegal-1993, Kenya-1998, and Romania 1996, as well as to the recent human isolate from Volgograd-1999. It is distinguishable from the group including both the New York-1999 and Israel-1998 isolates, as well as a WN virus recently isolated in our laboratory from the brain of a human fatal case that occurred during an outbreak in the governorates of Mahdia and Sfax on the Tunisian coast in 1997 (H. Triki, unpub. data).

### General Survey in Horses

To determine the number of infected horses and thus the number of asymptomatic infections, a serosurvey study has been undertaken, which includes all horses located within a 10-km radius of confirmed cases. A total of 5,133 sera were collected from September to November 2000 from the three provinces where cases were reported (Herault, Gard, and Bouches du Rhone). Preliminary results showed 428 (8.3%) horses with IgG antibodies; 248 had IgM antibodies. Analysis of these data is in progress, especially to determine rates of seropositivity for each commune. (A commune is the smallest French administrative subdivision, which approximately corresponds to an English parish).

The geographic locations of the seropositive horses were compared with those of the clinically confirmed and probable cases (Figure 5).

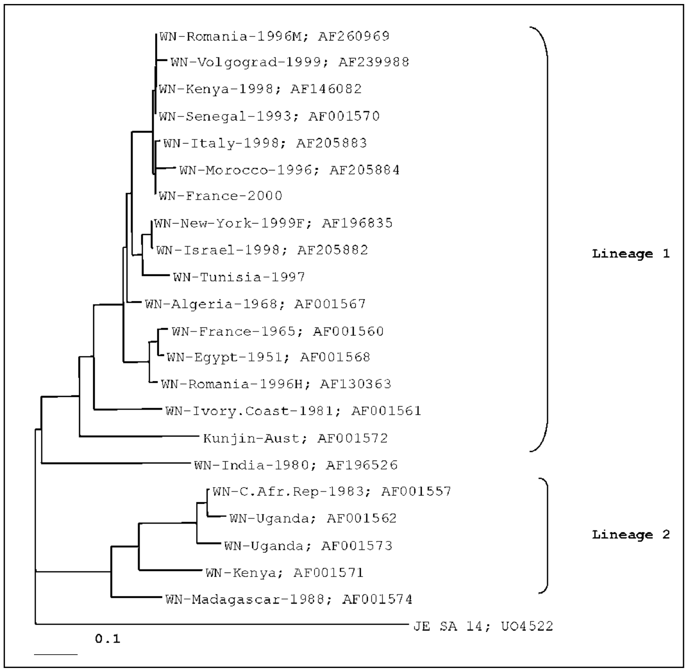

Figure 4. Phylogentic trees based on nucleic sequence data of E-glycoprotein gene fragments of 254 bp.*
*GenBank accession numbers for the sequences included in the tree are indicated.

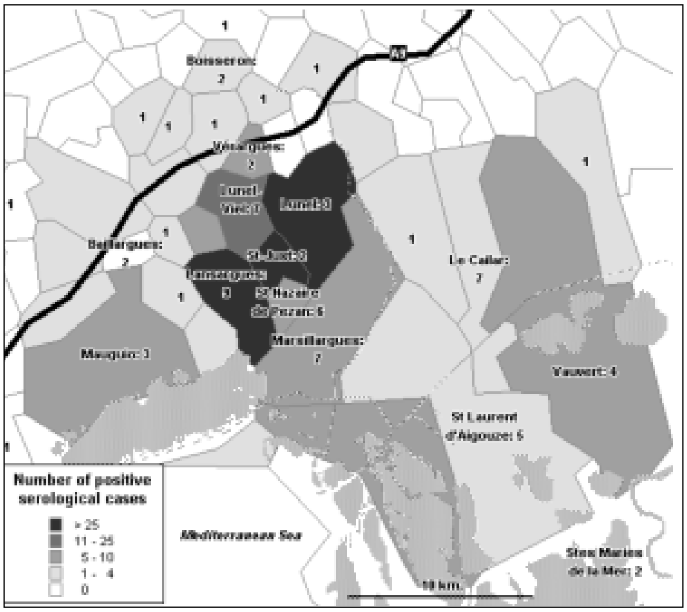

Figure 5. Geographic location of confirmed and probable clinical cases and serologically positive cases (according to serosurvey) of West Nile infection in equines, France. Data are grouped by commune, the boundaries of which are indicated. (The commune is the smallest French administrative subdivision, which approximately corresponds to an English parish). Numbers indicate clinical cases. Of the 76 cases, 73 are shown; the rest occurred more than 10 km outside the area. Names of communes in which more than one clinical case occurred are indicated. For each commune, the color (see key) indicates the number of positive serologic cases. The first cases were reported in Lansargues.

### Discussion

In this report we described the 2000 WN outbreak in horses in southern France. Only a few reports of WN virus encephalomyelitis cases in equines have been published. In Egypt, a high prevalence of WN antibodies (54%) was observed during a serologic survey conducted from January to May 1959 in 436 equines (horses, donkeys, and mules). One suspected case was fatal and confirmed by viral isolation from the brain (25). In France during the 1962 WN outbreak in Camargue, several horses were suspected to be infected (6). In Morocco (provinces of Kenitra and Larache), 94 equines were affected from August to mid-October 1996; 42 died. The disease was reported in all age categories (9), and virus was isolated from a brain biopsy sample (10). In Italy, from August to beginning October 1998, 14 horses in Tuscany had laboratory-confirmed WN virus infection, and 6 animals died (12). Virus was isolated from a brain biopsy sample (V. Deubel, unpub. data). In Israel in 1998, 18 serum samples from horses with encephalomyelitis had WN-neutralizing antibodies, and virus was isolated from the brain of a stork (26). In 1999, thousands of geese were destroyed when WN virus was identified in commercial flocks (27). In the northeastern United States, 20 horses were infected by WN virus in 1999; 9 died (28). In 2000, 63 equine cases, approximately 35% fatal, were confirmed. The first case was identified in mid-August 2000 (29).

In France, the outbreak started in August 2000 and ended in November. Most positive samples (80%) were obtained before September 30. The death rate during this outbreak (28%) was lower than observed in Morocco (45%), Italy (43%), and North America (45% and 35%). Most fatal cases (57.1%) occurred at the beginning of the outbreak, before September 15. At that time, most veterinary practitioners thought that the disease could not be cured and did not apply symptomatic treatment.

Horses, as well as humans, are generally considered to be dead-end hosts of WN infection; however, little is known about the duration and magnitude of viremia. Experimental infections in horses and donkeys in Egypt (25) and in France (30,31) showed undetectable or low viremia of short duration. However, these experiments were conducted in different conditions with different WN strains; therefore, it is difficult to extrapolate from these results.

During the present outbreak, we were unable to detect WN virus (by RT-PCR and inoculation into cell culture) in the blood of a few animals tested, including animals with virus identified in the brain. In contrast, using intracerebral inoculation into mice, virus was isolated from blood samples during previous epidemics in Egypt, Israel, South Africa, and France.

During the French outbreak, WN IgM antibodies were not detected in 18 IgG-positive animals with neurologic signs from which blood samples were obtained. In the absence of a second blood sample and in the context of the outbreak, we concluded that these animals had a recent WN virus infection. This conclusion was supported by previous serosurveys conducted in 1999 and 2000 that demonstrated absence of *Flavivirus* antibodies in horses (unpub. data). These data suggest that in some cases IgM response was very low or nondetectable by commonly used techniques. This may have implications for the diagnosis of recent infections.

The outbreak appeared limited to a restricted area within a 15-km radius. This region, near Camargue National Park, where the 1962 outbreak occurred, is characterized by its original flora (wetlands, rice fields, garriguea [a geographical dry area, typical of the mediterranean basin]) and fauna (more than 300 migratory and resident bird species and large populations of mosquitoes). No abnormal deaths of birds were reported. An epidemiologic investigation of WN virus in birds, including five common nesting species, is in progress. The vector(s) involved in the present outbreak is (are) still unknown.

Thousands of horses live in this region. Thus, our large serosurvey should allow us to precisely determine the geographic distribution of the infection and the number of asymptomatic infections in the geographic area where the outbreak occurred.

Two gamekeepers had WN-neutralizing antibodies, and one of them also had IgM antibodies. Thus, human transmission occurred during this outbreak. However, in the absence of a serologic survey, evaluating the level of human infection among persons living in the infected area is not possible.

The main concern for 2001 is the possibility of persistence of virus transmission and thus the risk of human infections. Natural vertical transmission of WN virus in *Culex* mosquitoes (32) or survival in overwintering mosquitoes (3,33) could explain the persistence of the virus. However, human transmission likely depends on several factors, including environmental factors, vectors, and amplifying host conditions. Usually in most countries where WN outbreaks have been documented, a few cases are reported during subsequent years (34).

Several questions about WN virus infection are still unresolved, among them whether WN infection is a major health problem for humans and horses. The main problem during this outbreak was not the disease itself but the economic consequences from the restricted movement measures imposed by the Commission of the European Communities (cancellation of horse exhibitions, layoffs of employees in equestrian centers). Gaining more knowledge about the role of horses in virus transmission under natural conditions is important.

### Acknowledgments

We are grateful to Ingrid Marendat and Jacques Labie for their technical assistance and to all the persons who contributed to the identification and follow-up of this outbreak.

Dr. Murgue is a researcher at Institut Pasteur and associate director of the National Center of References for Arboviruses and Viral Haemorrhagic Fever. Her main topics of research are dengue (clinical aspects and pathogenesis), West Nile virus, and other flaviviruses.

### References

1. Smithburn KC, Hughes TP, Burke AW, Paul JH. A neurotropic virus isolated from the blood of a native of Uganda. Am J Trop Med 1940;20:471-92.
2. Zeller H. West Nile: une arbovirose migrante d'actualité. Med Trop 1999;59:490-4.
3. Taylor RM, Work TH, Hurlbut HS, Rizk F. A study of the ecology of West Nile virus in Egypt. Am J Trop Med 1956;5:579-620.
4. Bernkopf H, Levine S, Nerson R. Isolation of West Nile virus in Israel. J Infect Dis 1953;93:207-18.
5. Panthier R, Hannoun C, Beytout D, Mouchet J. Epidémiologie du virus West Nile: étude d'un foyer en Camargue. III. Les maladies humaines. Annales de l'Institut Pasteur 1968;115:435-45.
6. Joubert L, Oudar J, Hannoun C, Beytout D, Corniou B, Guillon et al. Epidémiologie du virus West Nile: étude d'un foyer en Camargue. IV. La méningo-encéphalomyélite du cheval. Annales de l'Institut Pasteur 1970;118:239-47.
7. McIntosh BM, Jupp PG, Dos Santos, Meenehan GM. Epidemics of West Nile and Sindbis viruses in South Africa with *Culex (Culex) univittatus* Theobald as vector. S Afr J Med Sci 1976;72:295-300.
8. Le Guenno B, Bougermouth A, Azzam T, Bouakaz R West Nile: a deadly virus? Lancet 1996;348:1315.
9. Tber Abdelhaq A. West Nile fever in horses in Morocco. Bulletin de l'Office International des Epizooties 1996;11:867-9.
10. El Harrack M, Le Guenno B, Gounon P. Isolement du virus West Nile au Maroc. Virologie 1997;1:248-9.
11. Tsai TF, Popovici F, Cernescu C, Campbell GL, Nedelcu NI. West Nile encephalitis in southeastern Romania. Lancet 1998;352:767-71.
12. Cantile C, Di Guardo G, Eleni C, Arispici M. Clinical and neuropathological features of West Nile virus equine encephalomyelitis in Italy. Equine Vet J 2000;32:31-5.
13. Platonov AE, Shipulin GA, Shipulina AY, Tyutyunnik EN, Frolochkina TI, Lanciotti RS, et al. Outbreak of West Nile virus infection, Volgograd region, Russia, 1999. Emerg Infect Dis 2001;7:128-32.
14. Siegel-Itzkovich J. Twelve die of West Nile virus in Israel. BMJ 2000;321:724.
15. Rappole JH, Derrickson SR, Hubalek Z. Migratory birds and spread of West Nile virus in the Western Hemisphere. Emerg Infect Dis 2000;6:319-27.
16. Novello AC. West Nile virus in New York State: the 1999 outbreak and response plan for 2000. Viral Immunol 2000;4:463-7.
17. Hubalek Z, Halouzka J, Juricova Z. West Nile fever--a reemerging mosquito-borne viral disease in Europe. Emerg Infect Dis 1999;5:594-5.
18. Panthier R. Epidémiologie du virus West Nile: étude d'un foyer en Camargue. I. Introduction. Annales de l'Institute Pasteur 1968;114:519-20.
19. Hannoun C, Panthier R, Mouchet J, Eouzan JP. Isolement en France du virus West Nile à partir de malades et du vecteur Culex modestus Ficalbi. C R Acad Sci 1964;259:4170-2.
20. Panthier R, Hannoun C, Oudar J, Beytout D, Corniou B, Joubert L, et al. Isolement du virus West Nile chez un cheval de Camargue atteint d'encéphalomyélite. C R Acad Sci 1966;262:1308-10.
21. Rollin PE, Rollin D, Martin P, Baylet R, Rodhain F, Hannoun C. Résultats d'enquêtes séroépidémiologiques récentes sur les arboviroses en Camargue: populations humaines, equines, bovines et aviaries. Medecine et Maladies Infectieuses 1982;12:77-80.
22. Karabatsos N, editor. International catalogue of arboviruses, including certain other viruses of vertebrates. 3rd ed., and Supplements 1986-98. San Antonio: American Society of Tropical Medicine and Hygiene; 1985. p. 977.
23. Zeller HG, Fontenille D, Traore-Lamizana M, Thiongane Y, Digoutte JP. Enzootic activity of Rift valley Fever virus in Senegal. Am J Trop Med Hyg 1997;56:265-72.
24. Berthet FX, Zeller HG, Drouet MT, Rauzier J, Digoutte JP, Deubel V. Extensive nucleotide changes and deletions within the envelope glycoprotein gene of Euro-African West Nile viruses. J Gen Virol 1997;78:2293-7.
25. Schmidt JR, El Mansoury HK. Natural and experimental infection of Egyptian equine with West Nile virus. Ann Trop Med Parasitol 1963;57:415-27.
26. Malkinson M, Banet C, Weisman Y. Intercontinental spread of West Nile virus by wild birds--recent epidemiological findings in Israeli livestock and birds. In: Proceedings of the 2nd International Conference on Emerging Zoonoses, Strasbourg, France, 1998.
27. Office International des Epizooties. West Nile fever in Israel in geese. Disease Information 99;12:166.
28. Office International des Epizooties. West Nile fever in the United States of America: in horses. Disease Information 1999;12:150-1.
29. Centers for Disease Control and Prevention. Update: West Nile virus activity--eastern United States, 2000. MMWR Morb Mortal Wkly Rep 2000;49:1044-7.
30. Oudar J, Joubert L, Lapras M, Guillon JC. Reproduction expérimentale de la méningo-encéphalomyélite du cheval par l'arbovirus West Nile. II. Etude anatomo-clinique. Bull Acad Vét 1971;Tome XLIV:147-58.
31. Joubert L, Oudar J, Hannoun C, Chippaux M. Reproduction expérimentale de la méningo-encéphalomyélite du cheval par l'arbovirus West Nile. III. Relations entre la virologie, la sérologie et l'évolution anatomo-clinique. Conséquences épidémiologiques et prophylactiques. Bull Acad Vét 1971;Tome XLIV:159-67.
32. Miller BR, Nasci RS, Godsey MS, Savage HM, Lutwama JJ, Lanciotti RS, et al. First field evidence for natural vertical transmission of West Nile virus in *Culex Univittatus* complex mosquitoes from Rift Valley province, Kenya. Am J Trop Med Hyg 2000;62:240-6.
33. Centers for Disease Control and Prevention. Update: surveillance for West Nile virus in overwintering mosquitoes--New York. MMWR Morb Mortal Wkly Rep 2000;49:178-9.
34. Cernescu C, Nedelcu N, Tardei G, Ruta S, Tsai TF. Continued transmission of West Nile virus to humans in southeastern Romania, 1997-1998. J Infect Dis 2000;181:710-2.
